# Circulating tumor DNA in cancer diagnosis, monitoring, and prognosis

**DOI:** 10.1186/s43046-022-00109-4

**Published:** 2022-02-21

**Authors:** Sudeepto Saha, Yusha Araf, Salman Khan Promon

**Affiliations:** 1 Department of Life Sciences, School of Environment and Life Sciences, Independent University, Bangladesh (IUB), Dhaka, Bangladesh; 2 Department of Genetic Engineering and Biotechnology, School of Life Sciences, Shahjalal University of Science and Technology, Sylhet, Bangladesh

**Keywords:** Circulating tumor DNA (ctDNA), Cancer biomarkers, Cancer diagnosis

## Abstract

**Background:**

Circulating tumor DNA (ctDNA) has become one of the crucial components for cancer detection with the increase of precision medicine practice. ctDNA has great potential as a blood-based biomarker for the detection and treatment of cancer in its early stages. The purpose of this article was to discuss ctDNA and how it can be utilized to detect cancer. The benefits and drawbacks of this cancer detection technology, as well as the field’s future possibilities in various cancer management scenarios, are discussed.

**Main text:**

ctDNA has clinical applications in disease diagnosis and monitoring. It can be used to identify mutations of interest and genetic heterogeneity. Another use of ctDNA is to monitor the effects of therapy by detecting mutation-driven resistance. Different technologies are being used for the detection of ctDNA. Next-generation sequencing, digital PCR, real-time PCR, and mass spectrometry are used. Using dPCR makes it possible to partition and analyze individual target sequences from a complex mixture. Mass-spectrometry technology enables accurate detection and quantification of ctDNA mutations at low frequency. Surface-enhanced Raman spectroscopy (SERS) and UltraSEEK are two systems based on this technology. There is no unified standard for detecting ctDNA as it exists in a low concentration in blood. As there is no defined approach, false positives occur in several methods due to inadequate sensitivities. Techniques used in ctDNA are costly and there is a limitation in clinical settings.

**Short conclusion:**

A detailed investigation is urgently needed to increase the test's accuracy and sensitivity. To find a standard marker for all forms of cancer DNA, more study is needed. Low concentrations of ctDNA in a sample require improved technology to provide the precision that low concentrations of ctDNA in a sample afford.

## Background

Over 8.2 million people die every year due to a lack of access to appropriate detection procedures [1]. As a result, it is always a challenge for researchers to look into methods to aid in cancer detection and monitoring. The available techniques have limitations and can be harmful to the patient's physical health during the diagnosis process. Radiology, for example, is a widely used method for cancer detection that employs ionizing radiation, which may pose health risks to the patient’s body [2]. On the other hand, practices such as ultrasound detection and MRI scans are ineffective for detecting minimal residual disease. Again, the “Solid Biopsy” method infiltrates the body and is incapable of monitoring dynamic changes in this process [3].

Because liquid biopsy is a technique for analyzing non-solid biological tissues, it has excellent potential to overcome the limitations of traditional methods. Cell-free DNA circulates in plasma and other bodily fluids such as urine and saliva. ctDNA is released when a tumor cell dies or through active secretion. ctDNA is a DNA fragment of approximately 166 base pairs [4]. Because ctDNA contains tumor-related genetics relevant to cancer development, it can be used as a biomarker.

There are a few strategies for detecting mutations using ctDNA, such as studying the loss of heterozygosity (LOH) [5, 6], DNA methylation, DNA integrity, and NGS, or detecting somatic alterations in patient plasma using digital PCR. Next-generation sequencing altered strategies by increasing understanding of genetic mutations and their identification in ctDNA. For example, in head and neck cancer, tumor suppressor genes such as TP53 and oncogenes such as EGFR and KRAS are mutations in non-small lung cancer [7, 8]. Not only can ctDNA be used for detection, but it can also be used to monitor response to cancer treatment and disease recurrence, as cancer antigen 15-3 (CA 15-3) is used as a marker in breast cancer treatment [9].

This article will look at several approaches to using ctDNA in the early detection of cancer. This paper also discusses the challenges, limitations, and potential applications of ctDNA.

## Main text

### Candidate analytes in liquid biopsy

With the popularization of precision medicine and personalized medicine, liquid biopsy has become a promising approach. In personalized medicine, therapies depend on an individual’s genetic mutation and gene expression analysis, which usually requires cancer tissue collection by biopsy. It is challenging to collect tumor tissue located in the deeper parts of the body and puts a burden on the patient. Liquid biopsy is an invasive method that solves that challenge by using different body fluids such as blood, urine, saliva, and serum as biomarkers. For cancer diagnosis, liquid biopsy uses other cancer-associated analytes, including circulating nucleic acids such as circulating tumor DNA (ctDNA), extracellular vesicles (EVs), extracellular RNA (exRNA), and circulating tumor cells (CTCs).

**Fig. 1:**
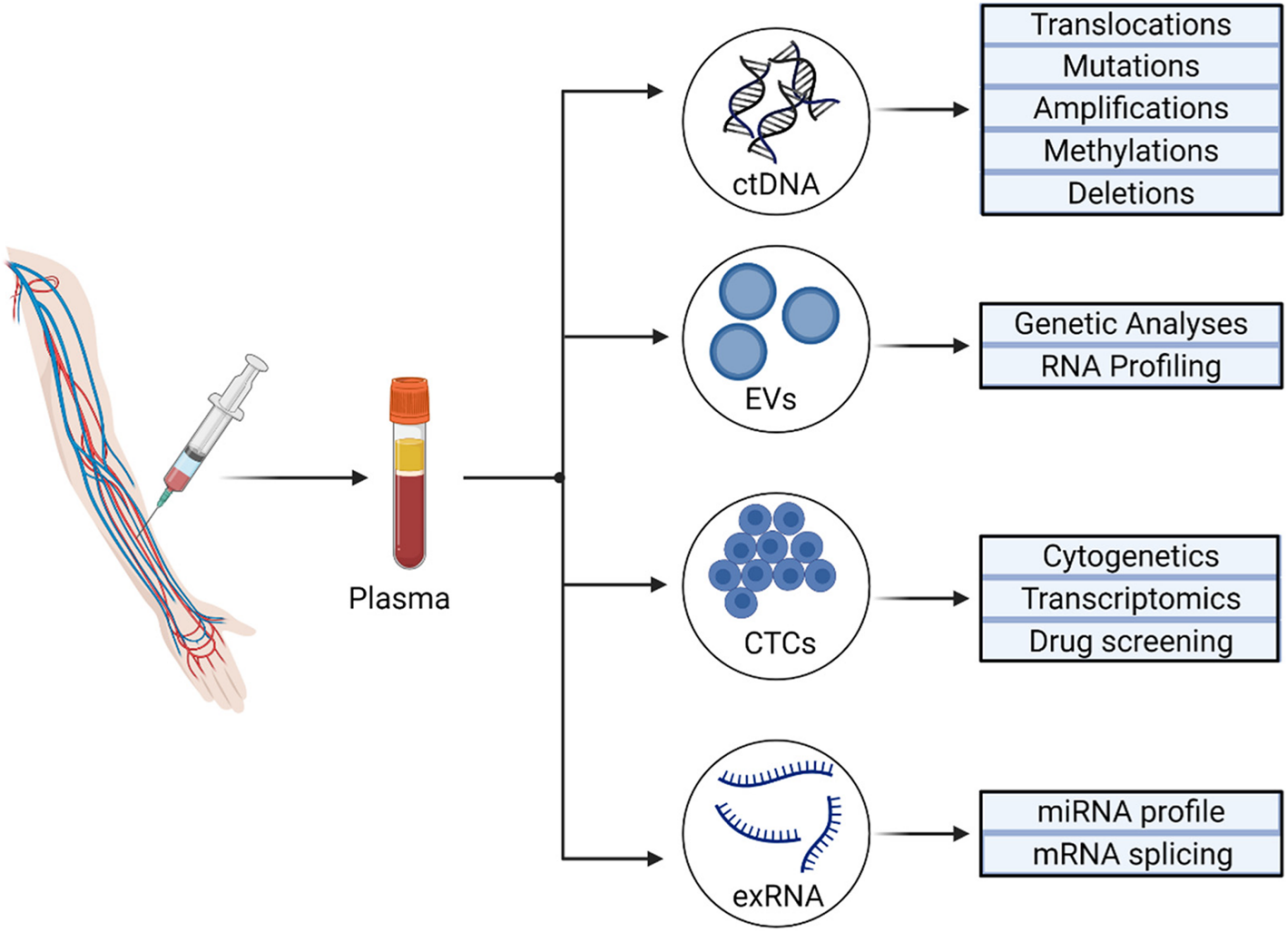
Candidate analytes in liquid biopsy and their possible usage in Cancer detection and prognosis

Nowadays, ctDNA and EVs are receiving increasing attention as novel analytes in liquid biopsy. EVs are lipid membranous vesicles released by almost all types of cells that play essential roles in cell-to-cell communication via horizontal transfer of cellular products like protein, RNA, lipids, and DNA fragments. The composition of EVs secreted by individual cell types varies depending on their origin. These phenomena are used to determine the presence and nature of cancer by using cancer-derived EVs [10]. CTCs are the cells derived from the solid tumor, and they are a crucial determinant of hematogenous metastasis and recurrence as they give information about cancer spreading. Circulating cell-free DNA (cfDNA) is a short fragment of nucleic acid found in body fluids, and one of the examples of cfDNA is circulating tumor DNA (ctDNA) derived from tumor cells. ctDNA harbors genetic and epigenetic changes present in the tumor, which can help predict treatment response and recurrence [11]. exRNA is the RNA found outside of cells in EVs or associated with platelets, lipoproteins, and protein complexes. As miRNA profiles are associated with cancer-specific conditions, non-EV-associated miRNAs or exRNA have potential uses as biomarkers [12] (Fig. 1).

### Biology of ctDNA

In precision medicine, ctDNA has introduced a highly efficient disease diagnosis system based on a patient’s genetic and epigenetic composition. ctDNA comprises fragmented DNA with a length of 166 base pairs [4] and is segregated from tumor cells at a low concentration. The fragmented DNA is wrapped around a single nucleosome. This nucleosome association protects ctDNA from the activity of blood-borne nucleases. This nucleosome association creates some opportunities. As an example, by deep sequencing and reading a map, it is possible to infer nucleosome occupation. And by determining the pattern of nucleosome occupation, we can estimate the origin of the tissue [13]. As ctDNA is a type of cfDNA, a challenging aspect of ctDNA analysis is often how little of the cfDNA is of tumor origin or ctDNA in a sample. Mutant allele frequency of mutations is used to measure the level of ctDNA, which is usually present in a patient’s tumor from above 25% to under 1 part in 10k [14].

### ctDNA as potential biomarkers for cancer

ctDNA as a biomarker has the potential to detect cancer at an early stage. The quantification study of ctDNA concentration showed that the level of ctDNA is higher in a malignant disease patient than in a healthy individual [15]. Another study showed that in breast, ovarian, lung, colorectal, and gastric cancers, ctDNA is associated with tumor volume, leading to shorter overall survival (OS) [16–18]. Some studies have presented contradictory results, indicating that concentration is not associated with overall or progression-free survival [19]. From those studies, it is possible to conceive the limitations of ctDNA for diagnostic purposes, but still, ctDNA can be used to monitor tumor progression.

ctDNA has clinical applications in disease diagnosis and monitoring. Some studies succeed in identifying mutations in a patient’s tumor by using ctDNA. For example, a PIK3CA mutation helped to identify breast cancer with 95% accuracy [20]. In lung cancer, gene mutations such as KRAS and EGFR, as well as various types of mutations in ovarian cancer, helped identify with 97% accuracy [21]. As a result, ctDNA has the potential to be used to identify mutations of interest and genetic heterogeneity.

Another use of ctDNA is to monitor the effects of therapy by detecting mutation-driven resistance [22]. A common factor in metastatic breast cancer patients is endocrine therapy resistance to the ESR1 mutation [23]. By using ctDNA, it is possible to achieve early detection of this kind of mutation and give treatment before clinical progression. Again, in gastrointestinal stromal cancer, it is possible to track the sensitivity to tyrosine kinase inhibitors (TKIs) in the patient’s body by tacking the mutation of KIT (S821F) and PDGFRA (D842V) [24]. This gives us an idea of how we can track cancer progression using serial genotyping in ctDNA (Table 1).

**Table 1:** List of cancer-specific biomarkers

Cancer type	Biological source	Markers	Potential application	Ref
Urinary cancer				
Prostate cancer	Plasma	TP53	Diagnosis	[25]
	Plasma	AR	Diagnosis/prognosis	[26]
	Plasma	HRR	Monitoring	[27]
Bladder cancer				
mUC	Plasma	FGFR3, ERBB2(HER2), ERCC2	Diagnosis	[28]
MIBC	Plasma	PIK3CA, TP53,TERT	Prognosis	[29]
Renal cancer				
ccRCC	Plasma	VHL, PBRM1, SETD2,BAP1	Monitoring	[30]
mRCC	Plasma	TP53, VHL, EGFR, NF1, ARID1A	Monitoring	[31]
pRCC	Plasma	MET	Monitoring	[31]
Female cancer				
Breast cancer	Plasma	ESR1, PIK3CA, TP53, MUC1	Monitoring	[9, 32]
Ovarian cancer	Plasma	KRAS, PIK3CA, BRAF, ERBB2	Monitoring	[33]
Digestive cancer				
Pancreatic cancer	Plasma	BRCA2, K-ras	Prognosis and monitoring	[34]
	Plasma	KDR, EGFR, ERBR2	Prognosis	[34]
Colorectal cancer	Plasma	KRAS, BRAF, APC, mSEPT9	Monitoring	[35]
Gastric cancer	Plasma	TP53, PIK3CA, FBXW7	Monitoring	[36]
Others				
Lung cancer				
NSCLC	Plasma	KRAS, LKB1, ROS1, MET	Diagnosis	[37]
	Plasma	EGFR, ALK	Diagnosis and monitoring	[37]
Melanoma	Plasma	BRAF, NRAS, TERT	Prognosis and monitoring	[38]

*mUC* metastatic urothelial carcinoma, *MIBC* muscle invasive bladder cancer, *ccRCC* clear cell renal cell carcinoma, *mRCC* metastatic renal cell carcinoma, *pRCC* papillary renal cell carcinoma, *NSCLC* non-small-cell lung carcinoma

### Detection technologies of ctDNA

Different technologies are being used for the detection of ctDNA. As ctDNA is fragmented DNA and the amount of ctDNA is as low as 0.01% of the total ctDNA, it makes the detection challenging, particularly in the early stages of tumor growth [21, 39]. A targeted approach can monitor the progression where a tumor-specific mutation will be tracked independent of the primary tumor. For example, different techniques like Q-PCR, BEAMing, Safe-SeqS, CAPP-Seq, and TAmSeq are used for such monitoring [40]. This type of targeted monitoring is extremely sensitive for low allele frequencies as low as 0.01%. Another strategy is whole-genome sequencing or whole-exome sequencing to find point mutations or copy number alterations (CNAs) [41]. This strategy allows us to find the novel changes, and information from the primary tumor is not needed. But it is pretty tricky as it requires a high concentration of ctDNA for reliable reconstruction of tumor-specific genome-wide changes. Currently, usage methods include next-genome sequencing, digital PCR, real-time PCR, and mass spectrometry technology.

NGS is one of the powerful tools for investigating rare mutations and detecting primary cancer diseases. In the era of precision medicine, the use of NGS has become widespread. NGS technology allows it to reflect therapeutic efficiency using ctDNA levels and find mutations in primary and metastatic lesions [22, 42]. Because of the low quantity of ctDNA, detection and analysis in this method are extremely difficult. It has an error rate of 0.1% to 1% depending on the applied platform [41]. However, as ctDNA isolation is minimally invasive and easily detectable in the early stages of tumor growth, NGS technology is rapidly developing. Digital PCR allows the detection of point mutations in ctDNA at low allele fractions. Using dPCR makes it possible to partition and analyze individual target sequences from a complex mixture to detect rare mutations. Droplet digital PCR (ddPCR) and BEAMing (beads, emulsions, amplification, and magnetics) are two typical dPCR platforms. The sensitivity of dPCR is determined by the number of individual compartments and individual sequences that can be created and analyzed, as well as the ease of use; different steps from sample injection to reaction analysis can be automated [43].

The real-time PCR-based method is another technique currently used, as this method is rapid amplification of nucleic acid. It also has a sensitivity of wild-type DNA of 10–20% allele frequency and almost no false-positive results. Several PCR-based techniques are available, among them allele-specific amplification (AS-PCR) and co-amplification at lower denaturation temperature (COLD-PCR), which have been used in routine clinical practice. COLD-PCR can detect single variations of about 0.1% and allows for the enrichment of this amount of a mutant allele to increase mutation detection sensitivity by up to 100-fold [44]. With those, this technique is cost-effective, which makes it a promising tool for detecting mutations. One of the drawbacks of most PCR-based methods is that their multiplexing ability is limited. Mass-spectrometry technology solves that limitation and enables accurate detection and quantification of ctDNA mutations at low frequency. Surface-enhanced Raman spectroscopy (SERS) and UltraSEEK are two systems based on mass-spectrometry technology. SERS is one of the ultrasensitive methods which been used to characterize tissues at the molecular level and provide specific spectroscopic compositions of tissues [45]. Again, UltraSEEK has a high multiplex capability and a rapid turnaround time of less than a day, and it needs a low input of DNA for a single analysis [46]. Those advantages make mass-spectrometry technology a promising technique for the detection and identification of circulating tumor DNA.

### Challenges and limitations in circulating DNA for early diagnosis

As tumor-specific mutations and methylations occur in ctDNA, it has excellent potential for the noninvasive detection of cancer. But for the diagnoses, predictive management, and guidance for the treatments of these cancers, accurate detection of specific cell-free nucleic acid is still not possible [47]. Also, biomarkers for different cancer are different, and familiar biomarkers have not been discovered yet.

ctDNA has not any unified standard for detection. Traditionally, a large amount of blood sample is necessary to detect ctDNA as it exists in a low concentration in blood [48]. In the detection process, the samples need to be purified. Unfortunately, the purification process needs to be improved. Tumor DNA is diluted with normal DNA in blood plasma which creates a challenge in subsequent analysis. As there is no defined approach, false positives occur in several methods due to inadequate sensitivities. Techniques used in ctDNA are costly, and there is a limitation in clinical settings.

### Future prospects and advantages of using ctDNA in clinical utility

Since there are some well-known challenges in repeatedly obtaining tissue biopsies, ctDNA can be an effective method for cancer detection. It can help overcome the inherent tumor heterogeneity of tissue biopsies for personalized therapy, and it can detect cancer earlier than imaging studies [49]. Though ctDNA has some limitations in clinical applications, it can detect food in real-time and is non-invasive and non-harmful. It also has the advantage of being highly sensitive and specific. When compared to other liquid biomarkers, such as protein biomarkers, ctDNA has the potential to be more informative, accurate, and precise [22]. It is also possible to monitor cancer by measuring ctDNA dynamics in the blood. It has promising prospects for using ctDNAs and methylation levels in ctDNAs to make diagnoses, prognoses, and treatment recommendations. As this is still a developing area of research, there is still room for advancement before it can be used routinely in clinical settings.

## Discussion and future perspectives

ctDNA is an emerging research area. Though it lacks clinical validity and utility, it has demonstrated high analytical performance as a biomarker. With ctDNA, it is possible to measure and monitor cancer dynamics with high specificity and sensitivity. There is still a significant need for significant progress in developing sensitive technologies that allow for the detection and analysis of tumor material in body fluids. Because ctDNA detection indicates a significant tumor mutational status, it holds great promise for future cancer progression and drug response monitoring. However, because ctDNA represents only a minor fraction of total cfDNA, detecting low allele variants reliably and reproducibly is difficult. However, with more research and technological advancement, ctDNA could become a weapon for early cancer detection.

### Exclusive summary

Cancer detection is a problem in cancer treatment because cancer prognosis is dependent on detectable primary tumors. The tumor tissue biopsy is primarily used to obtain mutation-related information after the primary tumor has been detected. However, there are a few drawbacks to this method. To begin with, obtaining tumor tissue from a different type of cancer for biopsy is difficult. Second, tumor resistance to any treatments must be monitored, which is impossible to do with tumor tissue biopsy. As a result, ctDNA can be thought of as a more practical method of monitoring patients in real time. ctDNA is a noninvasive biomarker that could be used to provide diagnostic and prognostic information during and after treatment. It could be used as a precision medicine biomarker to detect tumor heterogeneity and clonal selection. Not only that, but ctDNA can also reveal DNA mutations, epigenetic changes, and other tumor-specific abnormalities like microsatellite instability (MSI) and loss of heterozygosity (LOH). In addition, ctDNA has some drawbacks, such as false-positive results. As a result, it has a limit as a biomarker for early detection. Though ctDNA studies revealed inconsistencies in detecting ctDNA mutations, they also demonstrated the potential for use in systematic therapy. By overcoming the major challenges of ctDNA analysis, assay sensitivity, and specificity, ctDNA can be used as a tool. Already, ctDNA-based biomarkers for cancer detection and monitoring have been discovered. The development of accurate detection methods based on ctDNAs could benefit cancer patient treatments, and with an effective cancer diagnosis, clinical outcomes can be improved quickly.

## Conclusions

Complete validation in clinical trials is currently required to increase the clinical utility of ctDNA as a liquid biopsy. There is an urgent need for a comprehensive study to improve the test’s accuracy and sensitivity. Additional research is needed to find a standard marker for all types of cancer DNA, and technologies must improve to allow for the precision that low concentrations of ctDNA in a sample provide. Cancer treatment and diagnosis in the future can have a big impact on ctDNA collected from liquid biopsies.

## Data Availability

Not applicable.

## Abbreviations

ctDNA: Circulating tumor DNA
MSI: Microsatellite Instability
CNAs: Copy number alterations
LOH: Loss of heterozygosity
EVs: Extracellular vesicles
exRNA: Extracellular RNA
CTCs: Circulating tumor cells
